# Resveratrol ameliorates hepatic steatosis and inflammation in methionine/choline-deficient diet-induced steatohepatitis through regulating autophagy

**DOI:** 10.1186/s12944-015-0139-6

**Published:** 2015-10-24

**Authors:** Guiyuan Ji, Yuqi Wang, Yingxun Deng, Xin Li, Zhuoqin Jiang

**Affiliations:** Guangdong Provincial Institute of Public Health, Guangdong Provincial Centre for Disease Control and Prevention, Guangzhou, 511430 China; Guangdong Provincial Key Laboratory of Food, Nutrition and Health, School of Public Health, Sun Yat-sen University, Guangzhou, 510080 China; Institute of Toxicology, Guangdong Provincial Centre for Disease Control and Prevention, Guangzhou, 511430 China

**Keywords:** Resveratrol, Methionine/choline-deficient, Non-alcoholic steatohepatitis, Autophagy, Hepatic steatosis, Hepatic inflammation, TBARS, TNF-α, IL-1β, IL-6

## Abstract

**Background:**

Non-alcoholic steatohepatitis (NASH) is one of the leading causes of chronic liver disease that can progress to liver fibrosis, cirrhosis and eventually hepatocellular carcinoma. Resveratrol, a naturally occurring phytoalexin, is believed to have therapeutic effects on hepatic steatosis. However, the effect of resveratrol on NASH and the underlying mechanism is not fully illustrated. In the present study, we aimed to exam the effect of resveratrol on methionine/choline-deficient (MCD) diet or medium-induced hepatic steatosis, oxidation and inflammation, and to explore the possible mechanism.

**Methods:**

C57BL/6 mice and AML12 cells were treated with MCD alone or in combination with different concentrations of resveratrol (100 mg/kg/day or 250 mg/kg/day for mice and 25 μmol/L, 50 μmol/L, or 100 μmol/L for cells). Levels of aminotransferases (ALT), interleukin 1β (IL-1β), IL-6, and tumor necrosis factor alpha (TNF-α) were measured, concentrations of triglyceride (TG) and thiobarbituric acid reactive substances (TBARs) were determined, and expressions of proteins involved in autophagy were analyzed.

**Results:**

The results indicate that MCD diet or medium induced NASH in mouse and AML12 cell, which was confirmed by the elevated levels of TG, TNF-α, IL-1β, IL-6, ALT and TBARS in mice serum or cell culture medium. Resveratrol administration slowed down NASH progression, decreased the levels of ALT, TG, TBARS, IL-1β, IL-6, downregulated mRNA expressions of TNF-α, IL-1β, IL-6, and regulated the expressions of proteins involved in autophagy, both *in vitro* and *in vivo*. However, an autophagical inhibitor significantly impaired the protective role of resveratrol on liver injury and inflammation.

**Conclusions:**

Resveratrol can attenuate hepatic steatosis and inflammation in MCD-induced NASH by regulating autophagy. Thus, resveratrol may be a promising agent for inhibiting lipid accumulation and inflammatory processes associated with NASH.

## Background

Non-alcoholic fatty liver disease (NAFLD) is described in the 60 % of the subjects with hyperlipidemia, and in 83 % of those with both elevated serum alanine aminotransferase (ALT) and hyperlipidemia [1, 2]. It is reported that 10 ~ 25 % of NAFLD subjects develop NASH [3]. Apart from significant hepatic lipid accumulation, NASH is also clinically characterized by histopathologic abnormalities, hepatocellular injury, as well as systemic and hepatic inflammation [4, 5]. Currently, there are no approved treatment strategies for NASH supported by systematic and valid studies. However, some phytonutrients or phytochemicals, including anthocyanid [6], soy isoflavones [7] and black rice extract [8], have received increasing attentions even though the specific mechanisms are not clearly identified.

Resveratrol (3,4,5-trihydroxystibene, Res), a naturally occurring polyphenol, is a phytoalexin found in a wide variety of plants such as mulberry, Japanese knotweed, peanuts and grapes [9]. Resveratrol has been implicated in the protection against inflammation [10], hypercholesterolemia [11], aging and cancer [9, 12]. It was reported that resveratrol treatment could inhibit hepatic satellite cell activation and improve hepatic endothelial dysfunction in cirrhotic rats [13], and resveratrol influences metastasis of other primary cancers in the liver [14]. Although several investigations have reported that resveratrol could alleviate hepatic steatosis [15–17], other researchers lead to contradictionary conclusions [18]. Few studies focused on the relationship between resveratrol and steatohepatities, and the exact effect and the possible mechanism remains elusive.

In the present study, we aimed to investigate and evaluate the potential effects of resveratrol on methionine/choline-deficient (MCD) diet or medium-induced NASH, and to explore the possible mechanism.

## Results

### Effects of resveratrol on lipid accumulation in MCD-induced NASH

**Fig. 1:**
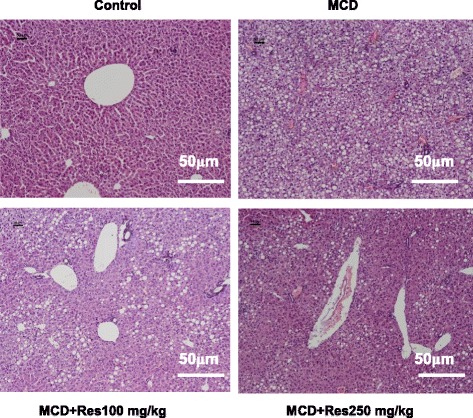
Histological analysis of mice livers. Mice were fed with normal chow diet (Control), with MCD diet (MCD), with MCD diet and treated P.O with 100 mg/kg resveratrol (MCD + Res 100), with MCD diet and treated P.O with 250 mg/kg resveratrol (MCD + Res 250) for 4 weeks. Their liver tissue sections were stained with H&E and examined under a light microscope. Data shown are representative images (magnification 20x) of each group (*n* = 10 per group) and all mice were analyzed simultaneously

All the animals tolerated the experimental procedures well and no deaths occurred during the 4 week study. No significant difference was observed in the baseline of mice body weights (data not shown). As shown in Table 1, four weeks of MCD diet feeding dramatically decreased body weight and liver weight (*P* < 0.01, *P* < 0.05) but increased liver/body weight ratio (*P* < 0.01), while resveratrol intervention increased body weight but caused a moderate decrease in the liver/body weight ratio. In addition, the average calorie intake showed no significant difference between the resveratrol untreated mice and the resveratrol treated mice (data not shown). Hepatic histopathology is the golden standard in NASH diagnosis. As shown in Fig. 1, liver tissue in the MCD-fed mice displayed different sizes of lipid droplets in the cytoplasm, and inflammatory infiltration. However, lipid accumulation and inflammatory infiltration were decreased in the resveratrol treated mice. The result of quantified NAFLD Activity Score (NAS) showed MCD diet lead to elevated NAS, while resveratrol intervention suppressed MCD-induced NAS increase. Similar result was found in the liver TG content assay. There were significant increases in triglyceride levels in MCD-fed mice compared with control mice, while resveratrol administration obviously inhibited MCD diet-induced TG increase (Table 1).

**Table 1:** Resveratrol reduces the severity of NASH induced by MCD

	Control	MCD	MCD + R-L	MCD + R-M	MCD + R-H
Mice (In vivo)					
Final body weight (g)	26.5 ± 1.6	13.7 ± 0.8^**^	14.2 ± 0.8		15.2 ± 0.8
Liver weight (g)	1.1 ± 0.1	0.8 ± 0.1^*^	0.7 ± 0.1		0.6 ± 0.1
Liver/body (%)	2.5 ± 0.3	9.5 ± 1.3^**^	6.1 ± 0.4		5.3 ± 0.7^***^
Liver TG (μg/mg Pro)	2.1 ± 0.3	10.3 ± 1.6^**^	7.2 ± 0.8^***^		3.6 ± 0.6^****^
Serum ALT (U/L)	57.2 ± 8.3	174.1 ± 32.8^**^	125.0 ± 21.7^***^		96.4 ± 18.1^****^
Liver TBARs (μmol/g Pro)	2.1 ± 0.3	10.3 ± 1.5^*^	7.6 ± 0.9^***^		5.21 ± 0.6^****^
NAS score	2.0 ± 0.3	8.7 ± 1.2^*^	7.0 ± 0.5^***^		5.9 ± 0.4^****^
Cell (In vitro)					
ALT (U/L)	27.8 ± 3.8	78.9 ± 10.2^*^	66.2 ± 8.7	49.1 ± 6.4^***^	40.3 ± 6.5^****^
AST (U/L)	34.6 ± 3.0	95.8 ± 11.3^*^	83.7 ± 9.8	67.0 ± 7.3^***^	52.9 ± 7.0^****^
TBARs (μmol/g Pro)	13.1 ± 1.2	41.7 ± 5.8^**^	37.4 ± 3.8	29.8 ± 2.8^***^	20.5 ± 2.3^****^
ROS	1.0 ± 0.2	5.6 ± 0.9^**^	3.9 ± 0.57^***^	2.7 ± 0.4^****^	2.1 ± 0.4^****^
TG (μg/mg Pro)	89.2 ± 8.5	276.3 ± 30.2^**^	190.5 ± 21.0^***^	143.1 ± 15.8^****^	95.4 ± 9.7^****^

Values are means ± SD, *n* = 10 (Serum and Liver) or *n* = 3 (Cell, three triplicate experiments)

*R* resveratrol, *MCD* methionine/choline-deficient diet or methionine/choline-deficient medium

*In vivo*, MCD + R-L (100 mg/kg), MCD + R-H (250 mg/kg)

*In vitro*, MCD + R-L (25 μmol/L), MCD + R-M (50 μmol/L), MCD + R-H (100 μmol/L)

^*^ *P* < 0.05, ^**^ *P* < 0.01 compared to Control

^***^ *P* < 0.05, ^****^ *P* < 0.01 compared to MCD

To evaluate the effects of MCD medium and resveratrol on cell viability of alpha mouse liver 12 (AML12), CCK-8 assay was conducted. AML12 cells were treated with MCD medium and 0, 1, 5, 25, 50, 100, or 200 μmol/L resveratrol for 12, 24, or 48 h. Cell counting kit (CCK-8) assay did not show any significant difference in viability of AML12 cells in the resveratrol-treated cells and untreated cells, suggesting that resveratrol is not cytotoxic. In this *in vitro* study, dosage of resveratrol was determined according to the cell viability test and results of a previous study [15]. AML12 cells were incubated with resveratrol (25 μmol/L, 50 μmol/L, or 100 μmol/L) in the presence or absence of MCD medium for 24 h. Cells were collected and TG content was determined. A similar trend of TG levels could also be found in AML12 cells, indicating that resveratrol may regulate the abnormal lipid accumulation in cellular NASH (Table 1).

### Effect of resveratrol on liver injury and oxidative stress in MCD-induced NASH

Aspartate transaminase (ALT) and alanine transaminase (AST) are generally determined to evaluate hepatocellular injury. Thiobarbituric acid reactive substances (TBARS) and reactive oxygen species (ROS) are markers of oxidative stress. The results indicate that MCD diet leads to an obvious increase in serum TBARs and ALT levels in NASH mice, while resveratrol treatment suppresses MCD diet-induced TBARs and ALT elevation.

AML12 cells were incubated with resveratrol (25 μmol/L, 50 μmol/L, or 100 μmol/L) in the presence or absence of MCD medium for 24 h. Levels of ALT and AST in cell culture medium were measured. The results showed that resveratrol could significantly decrease ALT and AST levels in MCD-treated cells. Additionally, resveratrol treatment significantly reversed the MCD-induced increase of TBARS and ROS (Table 1).

### Effect of resveratrol on the production of inflammatory cytokines in MCD-induced NASH

It is widely acknowledged that inflammation plays a vital role in the progression of hepatic steatosis to hepatic fibrosis and cirrhosis. In the present study, inflammatory cytokines in the serum or culture medium treated with MCD were measured.

Consistent with the results of hepatic biochemistry and histology, the MCD diet upregulated interleukin-1β (IL-1β), interleukin-6 (IL-6), tumor necrosis factor alpha (TNF-α) levels in serum, and increased their mRNA expression levels in liver. However, resveratrol administration significantly reduced their expressions in mice serum and liver (Table 2).

**Table 2:** Effects of resveratrol on inflammatory cytokine levels in MCD-induced NASH

	IL-1β		IL-6		TNF-α	
	Protein	mRNA	Protein	mRNA	Protein	mRNA
Serum or liver (*In vivo*)						
Control	24.7 ± 4.1	1.0 ± 0.3	19.2 ± 3.0	1.0 ± 0.3	31.8 ± 3.7	1.0 ± 0.3
MCD	216.9 ± 35.8^*^	5.9 ± 1.7^*^	124.7 ± 18.3^*^	11.7 ± 2.8^*^	198.4 ± 27.9^*^	7.8 ± 1.9^*^
MCD + R100	127.4 ± 15.4^**^	3.2 ± 0.8^**^	76.2 ± 7.7^**^	4.8 ± 1.2^**^	77.2 ± 8.15^**^	2.7 ± 0.7^***^
MCD + R250	69.1 ± 9.3^***^	1.0 ± 0.3^***^	43.1 ± 3.6^***^	2.2 ± 0.5^***^	54.8 ± 5.14^***^	1.4 ± 0.4^***^
Cell (*In vitro*)						
Control	28.4 ± 3.07	1.0 ± 0.1	86.2 ± 6.4	1.0 ± 0.2	117.4 ± 11.6	1.0 ± 0.1
MCD	86.9 ± 11.7^*^	2.8 ± 0.5^*^	278.5 ± 35.6^*^	5.4 ± 0.7^*^	786.5 ± 82.5^*^	9.1 ± 1.0^*^
MCD + R25	60.5 ± 8.6^**^	2.2 ± 0.4	149.2 ± 17.3^***^	4.6 ± 0.4	445.7 ± 40.3^**^	5.8 ± 0.5^**^
MCD + R50	47.2 ± 4.1^***^	1.7 ± 0.3^**^	124.7 ± 12.6^***^	3.3 ± 0.3^**^	360.1 ± 25.7^***^	3.6 ± 0.3^***^
MCD + R100	41.9 ± 3.9^***^	1.3 ± 0.1^***^	117.4 ± 10.2^***^	1.7 ± 0.3^***^	279.6 ± 16.3^***^	1.4 ± 0.2^***^

Values are means ± SD, *n* = 10 (serum) or *n* = 3 (medium, three triplicate experiments)

*MCD* methionine/choline-deficient, *R* resveratrol

^*^ *P* < 0.01 compared to Control. ^**^ *P* < 0.05, ^***^ *P* < 0.01 compared to MCD

We obtained similar results *in vitro* study. AML12 cells were incubated with resveratrol (25 μmol/L, 50 μmol/L, or 100 μmol/L) in the presence or absence of MCD medium for 24 h. ELISA analysis showed that levels of IL-1β, IL-6 and TNF-α were obviously increased in MCD-treated medium, while resveratrol administration inhibited MCD-induced elevation of these cytokines. The results of RT-PCR indicated that resveratrol could reduce MCD-induced increase in mRNA levels of IL-1β, IL-6 and TNF-α in AML12 cells (Table 2).

### Resveratrol attenuated hepatocyte lipid accumulation and inflammation in MCD-induced NASH partially by regulating autophagy

The above data showed that resveratrol could attenuate hepatocyte lipid accumulation and inflammation in MCD-induced NASH. The autophagy was expected to involve in the effect of resveratrol on MCD-treated cells or mice, thus expression levels of proteins (LC3-II and P62) involved in autophagy were determined by western blots analysis.

**Fig. 2:**
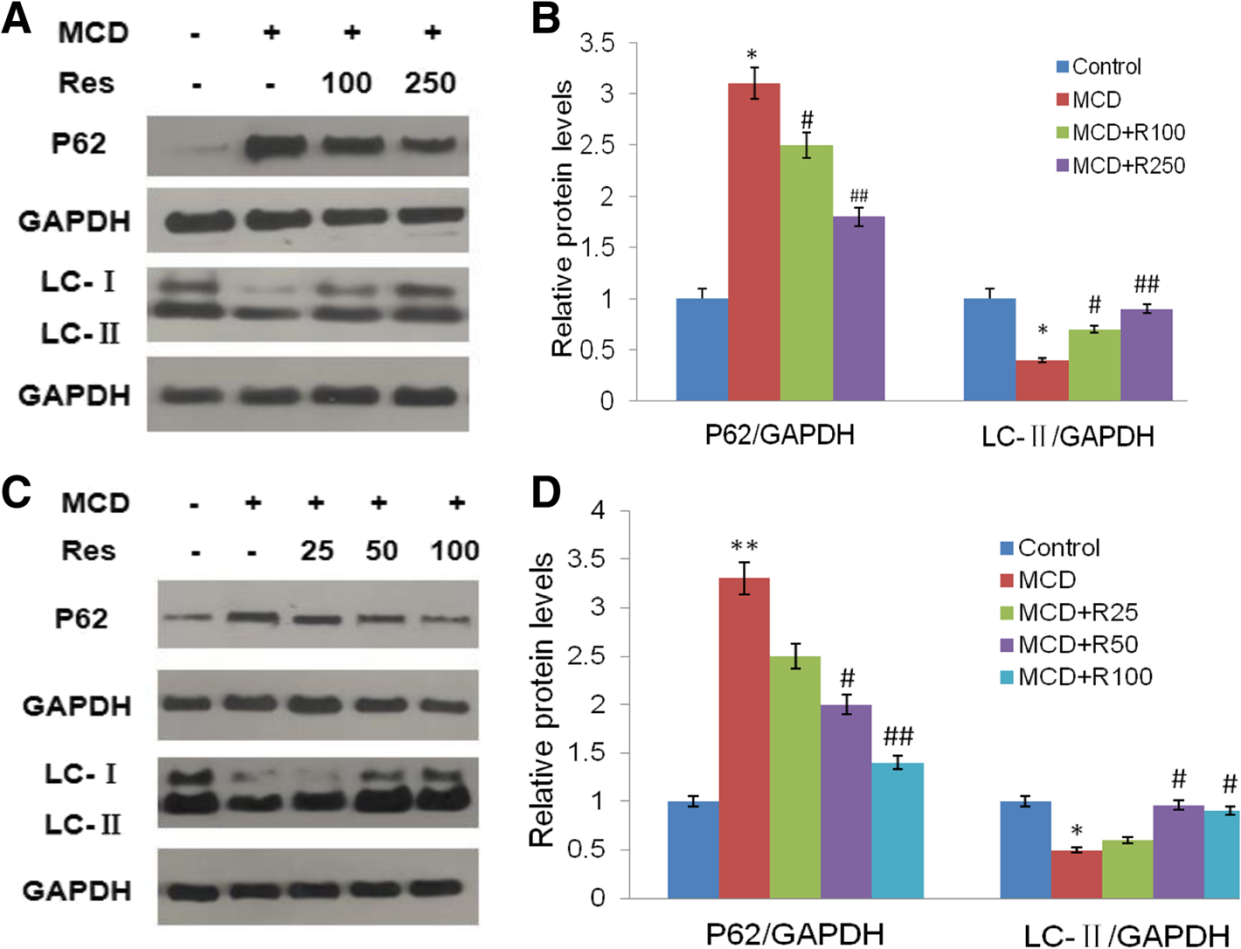
Effects of resveratrol on autophagy-related protein expressions in mice livers and AML12 cells. Mice were fed a normal diet (Control) or the MCD diet (MCD) with or without 100 mg/kg resveratrol (MCD + Res 100), 250 mg/kg resveratrol (MCD + Res 250) for 4 weeks. AML12 cells were incubated with resveratrol (25 μmol/L, 50 μmol/L, or 100 μmol/L) in the presence or absence of MCD medium for 24 h. LC3 and P62 levels were evaluated by immunoblotting both in mice livers (**a**,**b**) and in AML12 cells (**c**,**d**). Levels of LC3 and P62 were determined and the protein bands were quantified by densitometry and normalized to the amount of GAPDH in hepatic tissue and AML12 cells. The data were represented as mean ± SD. *P < 0.05, **P < 0.01 v.s. Control; ^#^ *P* < 0.05, ^##^ *P* < 0.01 v.s. MCD

As shown in Fig. 2, MCD diet or MCD medium significantly decreased LC3-II levels but increased P62 levels in mouse liver and AML12 cell, while resveratrol treatment increased LC3-II levels but decreased P62 expressions.

To further explore the role of autophagy in the resveratrol on MCD-induced NASH, an autophagy inhibitor, Chloroquine (CQ), was used in this study. The result indicated that CQ had no significant impact on cell viability in the presence of MCD medium or resveratrol, but CQ exacerbated MCD-induced increase in levels of ALT, AST, TG, TBARS and ROS. In accordance with the result shown in Table 2, resveratrol inhibited MCD-induced ALT, AST, TG, TBARS and ROS elevation, while these effects were attenuated by with CQ pretreatment (Table 3).

**Table 3:** Effects of chloroquine(CQ) intervention on resveratrol treatment in MCD medium induced-NASH

	Control	MCD	MCD + R	MCD + R + CQ	MCD + CQ
ALT(U/L)	29.5 ± 4.6	83.6 ± 6.4^*^	38.5 ± 5.9	57.6 ± 7.3^△△^	94.7 ± 11.8^▲▲^
AST(U/L)	32.9 ± 3.1	100.2 ± 10.5^*^	51.0 ± 6.4	73.0 ± 8.1^△△^	112.5 ± 12.4^▲^
TG (μg/mg Pro)	86.7 ± 9.2	294.5 ± 28.9^**^	139.8 ± 16.2	229.6 ± 21.5^△△^	381.0 ± 36.1^▲▲^
TBARs (μmol/g Pro)	12.1 ± 1.2	43.5 ± 4.7^**^	23.9 ± 2.4	27.1 ± 2.6^△^	53.6 ± 5.5^▲▲^
ROS	1.0 ± 0.2	6.6 ± 1.3^**^	2.4 ± 0.3	3.6 ± 0.7^△△^	9.8 ± 1.14^▲▲^
IL-1β (pg/ml)	25.1 ± 3.3	89.7 ± 12.3^**^	40.3 ± 4.3	68.3 ± 8.1^△△^	147.3 ± 13.3^▲▲^
IL-6 (pg/ml)	78.6 ± 5.8	285.5 ± 32.3^**^	185.1 ± 16.4	207.8 ± 21.6^△△^	358.6 ± 33.1^▲▲^
TNF-α (pg/ml)	121.3 ± 12.9	792.4 ± 82.7^**^	285.9 ± 25.7	541.3 ± 58.4^△△^	1043.9 ± 112.7^▲▲^
IL-1β mRNA	1.0 ± 0.1	3.2 ± 0.4^**^	1.3 ± 0.1	1.9 ± 0.2	7.4 ± 0.9^▲▲^
IL-6 mRNA	1.0 ± 0.2	5.7 ± 0.6^**^	2.2 ± 0.4	3.5 ± 0.4^△^	8.9 ± 1.1^▲▲^
TNF-α mRNA	1.0 ± 0.1	8.7 ± 0.9^**^	1.7 ± 0.3	3.6 ± 0.5^△^	14.3 ± 1.8^▲▲^

Values are means ± SD, *n* = 3; MCD: methionine/choline-deficient medium

*R* resveratrol (100 μmol/L), *CQ* chloroquine

^*^ *P* < 0.05, ^**^ *P* < 0.01 compared to Control

^△^ *P* < 0.05, ^△△^ *P* < 0.01 compared to MCD + R

^▲^ *P* < 0.05, ^▲▲^ *P* < 0.01 compared to MCD + R + CQ

Similar results were obtained with regards to the inflammation. The co-intervention of CQ and MCD medium significantly elevated the contents of inflammatory cytokines in cell culture medium, and increased their mRNA expressions in AML12 cells. Resveratrol administration could reverse MCD medium induced elevation of cytokine production, while the protective role of resveratrol on MCD-induced NASH was weakened by CQ (Table 3).

## Discussions

NASH has become the leading cause of elevated blood aminotransferase in the United States and other developed countries. However, there is no effective treatment for NASH. In the present study, we demonstrate that resveratrol administration partially reverses MCD-induced steatohepatitis partially via regulating autophagy *in vivo* and *in vitro*.

It is pretty common that high fat diet (HFD) was used to induce NASH, however, diet deficient in essential amino acids such as MCD is a well-established and widely-used nutritional model for inducing NASH, especially for an *in-vitro* study. In previous study, free fatty acid was used to induce NASH in vitro. However, free fatty acid treated cells showed lipid accumulation but no typical inflammation, thus it could not be used as an ideal NASH model in vitro study. MCD-induced NASH manifests multiple features of this disease in humans, particularly with respect to key histological and molecular alterations such as hepatic TG accumulation, metaflammation, and oxidative stress. Our study proves that by following the MCD diet, mice consistently developed hepatic steatosis, metaflammation and oxidative damage, which is in line with a previous report on this NASH [19]. Additionally, we also used MCD medium to induce cellular NASH model and most of the results agree well with the experiments performed in vitro.

The resveratrol dosage 100 mg/kg body weight (BW) and 250 mg/kg BW used in the mice study was fixed on the basis of the previous studies [20–22], in which the dosage of reveratrol were between 100 mg/kg BW and 200 mg/kg BW, however, some change was made because these dosage of reveratrol were applied in preventing or treating hepatic steatosis, which is the early stage of NASH. Therefore, a higher dosage (250 mg/kg BW) was applied in our present study.

The results showed that MCD diet or medium caused TG and TBARS elevation in mice and AML12 cells, while resveratrol administration significantly inhibited the elevation, which is in accordance with Scicchitano’s report [23]. The study indicated that resveratrol alleviated hyperlipidemia and lipid peroxidation, and reduced oxidative stress. Our present study indicated that resveratrol can attenuate hepatic steatosis, which was consistent with previous studies [15–17]. Current research on prevention and treatment of NASH by resveratrol intervention is very few, and some of these studies have reported contradictory results.

It is reported that resveratrol supplements improved inflammatory biomarkers in patients with NAFLD [24], and resveratrol decreased lipid deposition and inflammation in animal study [17, 25]. In our present study, we have obtained similar results while examining inflammation (Fig. 1 and Table 1). Another study, however, shows that the administration of 100 mg/kg resveratrol to Wistar rats fed with high fat diet for eight weeks does not lead to a significant anti-inflammatory effect [20]. Furthermore, some studies reported that resveratrol does not benefit patients or rats with NAFLD or NASH [18, 26, 27]. These contradictory conclusions may be due to different dosages, different rodent models and duration of resveratrol administration. Apart from alleviating lipid accumulation and inflammation, we also find that resveratrol has the ability to relieve oxidative stress in NASH (Table 1 and Table 2). Resveratrol administration obviously eliminated the oxidative stress triggered by MCD, which suggesting that resveratrol is protective and potentially inhibits the induction of proapoptotic or proinflammatory stress response cascades.

To further explore the possible mechanism, autophagy was investigated. Accumulating evidences suggest that autophagy is an important modulator of insulin resistance, lipid metabolism, metaflammation, and fibrosis, suggesting that autophagy may contribute to NAFLD progression [28, 29]. It is reported that resveratrol could induce autophagy under different circumstances [15, 30, 31]. In this study, autophagy related protein (LC3 and P62) were investigated. LC3-II is a specific marker of autophagy, and the LC3-II increases gradually when the isolated membrane (phagophore) is enclosed to form an autophagosome [32]. Under normal conditions, the constitutive autophagy degrades p62 and associated cargo (such as damaged proteins) from the cytoplasm [33]. As shown in Fig. 2, resveratrol administration increased the expression level of LC3-II but decreased the level of P62 in MCD-treated cells and mice, which indicated that resveratrol does induce autophagy in MCD-induced NASH. A previous study have reported that resveratrol could improve hepatic steatosis by inducing autophagy [15], which is different from our present study. Because hepatic steatosis and steatohepatities are two different stage in NAFLD, and the latter is a more serious stage.

It is reported that the efficacy of resveratrol on experimental NAFLD depends on severity of the pathology and timing of treatment [27]. Large-scale clinical trials and the therapeutic potential of resveratrol on NASH are needed in the future. The present study showed that autophagy inhibition cannot completely attenuate the effect of resveratrol on liver lipid accumulation and inflammation, thus indicating that other mechanism besides autophagy may participate in the protective effect of resveratrol on NASH.

## Conclusions

The present data indicated that resveratrol treatment can prevent and reverse hepatic lipid accumulation, oxidative stress and inflammation in MCD-induced NASH in vivo and in vitro, partially by regulating autophagy. This study provides evidence that resveratrol might be a promising innovative agent in preventing and treating NASH.

## Methods

### Animals and treatments

Forty male C57BL/6 mice (6 weeks old, 12 ± 2 g body weight) were purchased from the experimental animal center of Guangdong province, China. Mice were maintained under standard conditions of illumination (12-h light/dark cycle) and temperature (21-24 °C). Mouse model of MCD diet-induced NASH was duplicated according to previous studies [19, 34, 35].

After one-week adaptive feeding, 40 mice were randomly divided into normal control group, model control group, low and high dose of resveratrol intervention group. The mice in the normal control group (Control) got free access to the methionine/choline-supplement (MCS) diet (Trophic Animal Feed High-tech Co., China), mice in the model control group (MCD) were fed with MCD diet (Trophic Animal Feed High-tech Co., China), mice in the low-dose (M + R100) and high-dose (M + R250) resveratrol intervention group were fed with MCD diet and received intragastrical administration of resveratrol (100 mg/kg or 250 mg/kg body weight) daily. Mice in control and MCD group received the same volume of stroke-physiological saline solution gastrically. The treatment lasted 28 days. All experimental procedures were implemented in accordance with the Institutional Guidelines for Animal Experiments and all the protocols were approved by the animal experimental ethnics committee of the Sun Yat-sen University, China.

### Cell culture and treatments

The AML12 cell line was originally established from normal hepatocytes obtained from a CD1 male mouse strain, and the cells exhibit typical hepatocyte features such as peroxisomes and bile canalicular-like structures [36]. The AML12 cell line was an ideal model for NASH study in vivo [19]. AML12 cells (alpha mouse liver 12, ATCC #CRL-2254, Manassas, VA, USA) were cultured in Dulbecco’s modified eagle medium (DMEM, Gibco, Carlsbad, CA, USA) supplemented with 10 % fetal bovine serum (FBS, Gibco) and 1 % antibiotics in the 37 °C incubator with 5 % CO_2_. Cells were seeded at 1 × 10^6^ cells/ml in 100-mm dishes and were grown in either MCS medium or MCD medium.

Resveratrol was dissolved in dimethyl sulfoxide (DMSO) at a concentration of 100 mM and stored frozen in small aliquots and diluted at 1:1000 in culture medium. Control cells were treated with the vehicle (0.1 % DMSO). Chloroquine (10 mM) was prepared in 1× PBS and diluted with supplemented cell culture medium (1:1000) as needed, before cell exposure.

### Histological analysis

Liver tissue samples of each mouse were fixed in 10 % (v/v) neutral phosphate-buffered formalin (Sigma), dehydrated in a graded series of alcohol washes, cleared in toluene, and then embedded in paraffin blocks. Tissue sections were cut by a microtome and stained with hematoxylin and eosin (H&E) for histological analysis under light microscope.

NAFLD Activity Score (NAS), a tool to measure changes in NAFLD/NASH during therapeutic trials, was calculated by adding the scores of steatosis, lobular inflammation, and hepatocellular ballooning. Pathological features of steatosis (0–3), lobular inflammation (0–2), hepatocellular ballooning (0–2), and fibrosis (0–4) were scored by an experienced pathologist. Generally, NAS equal to or higher than 5 is a surrogate for the histological diagnosis of NASH [37].

### Cell viability assay

Cell viability was determined by Cell Counting Kit 8 (CCK-8, Dojindo, Japan) according to manufacturer’s instructions. Briefly, 1000 cells in 100 μL DMEM medium were seeded in 96-well plates. After 12 h incubation, the MCD medium and various concentrations of resveratrol (0, 1, 5, 25, 50, 100, or 200 μmol/L) were added, and the cells were incubated for another 12 h, 24 h or 48 h. CCK-8 solution (10 μL /well) was then added to the culture medium and incubated for an additional 3 h. Wells with medium alone (no cells) were served as blank controls and the absorbance of the solution was measured at 450 nm by a microplate reader.

### Biochemical analysis

Triglyceride (TG) contents in mice liver and AML12 cell were enzymatically measured by a commercially available test kit (Wako, Osaka, Japan) according to the instructions after extraction by sonication with a chloroform-methanol solution. Samples were then centrifuged for 5 min at 3000 rpm in 4 °C. Triglycerides in the upper layer were evaporated and dissolved in 0.5 mL of isopropyl alcohol. The absorption was measured at 600 nm and triglyceride concentrations were calculated. The levels of ALT in serum and cell cultured medium were determined by standard techniques (ALT Activity Assay kits, Cayman, Ann Arbor, USA). TBARs levels in mice liver and AML12 cell were measured by a TBARS assay kit (Cayman, Ann Arbor, USA).

### Enzyme-linked immunosorbent assay

Inflammatory cytokine levels in plasma and cell culture medium were measured using enzyme-linked immunosorbent assay (ELISA) kits specific for mouse IL-1, IL-6, TNF-α (MultiSciences LiankeBio, China), according to the manufacturer’s instructions. The plates were read at 450 nm and values at 570 nm were subtracted. Absorbance was converted to pg/mL for cytokine measurements in standard curves prepared with respective recombinant cytokines.

### Measurement of intracellular reactive oxygen species (ROS)

Intracellular ROS generation was determined by a ROS kit (Beyotime Institute of Biotechnology, China). AML12 cells were collected and washed twice with PBS and then fresh serum-medium containing 10 mM DCFH-DA (2′, 7′-dichlorofluorescin diacetate) was added to the cells. After 30 min incubation in the absence of light, cells were washed and then analyzed immediately by a flow cytometric assay (Beckman Coulter, Indianapolis, USA) with excitation and emission wavelengths of 488 and 525 nm, respectively.

### RNA extraction and reverse transcription polymerase chain reaction (RT-PCR)

Total RNA from mice livers or cultured cells was extracted using TRIzol reagent (Invitrogen, USA), according to the manufacturer’s instructions. Five micrograms of total RNA was synthesized into cDNA using a reverse transcriptase kit (Gene Copoeia, USA). The cDNA was then used as a template in PCR reactions using gene-specific primer pairs. Conventional PCR conditions using one primer pair per reaction tube were as follows: 95 °C for 1 min, 55 °C for 1 min, and 72 °C for 1 min for 40 cycles. After quantifying band intensities by using densitometry, the relative steady-state level of mRNA was calculated after normalizing to GAPDH. Primers specific for mice IL-1β, IL-6, TNF-α and GAPDH, for which sequences are shown in Table 4, were designed using the Primer 5.0 software design system and synthesized by Shanghai Invitrogen Biotechnology Company.

**Table 4:** Primer sequences used for amplification of cDNA by RT-PCR

Primer	Forward primer (5′-3′)	Reverse primer (5′-3′)	Accession No.
IL-1β	GAAATGCCACCTTTTGACAGTG	TGGATGCTCTCATCAGGACAG	NM_008361
IL-6	CTGCAAGAGACTTCCATCCAG	AGTGGTATAGACAGGTCTGTTGG	NM_031168
TNF-α	CAGGCGGTGCCTATGTCTC	CGATCACCCCGAAGTTCAGTAG	NM_013693
GAPDH	TGATGACATCAAGAAGGTGGTGAAG	TCCTTGGAGGCCATGTAGGCCAT	NM_008084

### Western blot analysis

Western blot analysis were performed to determine LC3B (D11) XP, P62 and GAPDH. Liver tissues or cultured AML12 cells were lysed at 4 °C in lysis buffer (Beyotime, China), sonicated, and then centrifuged 12,000 × g for 15 min at 4 °C, amples were collected using cytoplasmic protein extraction kits (Beyotime, China). Protein concentrations were determined by BCA protein assay kits. The total proteins (30 μg) were resolved by SDS-PAGE and then transferred to polyvinylidene difluoride membranes (Millipore, USA). The membrane was blotted with specific primary antibody and then secondary antibody. The bound enzymes were detected with Pierce enhanced chemiluminescence solution and the band intensity was quantified using a densitometric analysis program Quantity One (Bio-Rad, USA).

### Statistical analysis

All the data were expressed as a mean ± standard deviation (SD). Statistical analysis was undertaken using one-way ANOVA and the least significant difference (LSD). When ANOVA revealed significant differences, further analysis was performed using LSD for multiple comparisons. Differences between groups were considered statistically significant at *P* < 0.05. SPSS 16.0 computer program (SPSS Software, USA) was utilized for statistical analysis.

## Abbreviations

ALT: Alanine transaminase
AST: Aspartate aminotransferase
DMSO: Dimethyl sulfoxide
ELISA: Enzyme-linked immunosorbent assay
GAPDH: Glyceraldehyde 3-phosphate dehydrogenase
H&E: Hematoxylin and eosin
HCC: Hepatocellular carcinoma
IL-1β: Interleukin 1β
IL-6: Interleukin 6
MCD: Methionine/choline-deficient
MCS: Methionine/choline-supplement
NASH: Non-alcoholic steatohepatitis
NAFLD: Non-alcoholic fatty liver disease
NAS: NAFLD activity Score
ROS: Reactive oxygen species
Res: Resveratrol
RT-PCR: Reverse transcription polymerase chain reaction
TBARS: Thiobarbituric acid reactive substances
TG: Triglyceride
TNF-α: Tumor necrosis factor alpha

## References

[1] Browning JD (2006). Statins and hepatic steatosis: perspectives from the Dallas Heart Study. Hepatology.

[2] Mavrogiannaki AN, Migdalis IN. Nonalcoholic Fatty Liver Disease, Diabetes Mellitus and Cardiovascular Disease: Newer Data. Int J Endocrinol. 2013; doi:10.1155/2013/450639. 10.1155/2013/450639 PMC3638654 23653642

[3] Day CP (2005). Natural history of NAFLD: remarkably benign in the absence of cirrhosis. Gastroenterology.

[4] Tarantino G, Savastano S, Colao A (2010). Hepatic steatosis, low-grade chronic inflammation and hormone/growth factor/adipokine imbalance. World J Gastroenterol.

[5] Asrih M, Jornayvaz FR (2013). Inflammation as a potential link between nonalcoholic fatty liver disease and insulin resistance. J Endocrinol.

[6] Guo H, Xia M, Zou T, Ling W, Zhong R, Zhang W (2012). Cyanidin 3-glucoside attenuates obesity-associated insulin resistance and hepatic steatosis in high-fat diet-fed and db/db mice via the transcription factor FoxO1. J Nutr Biochem.

[7] Xiao CW, Wood CM, Weber D, Aziz SA, Mehta R, Griffin P (2014). Dietary supplementation with soy isoflavones or replacement with soy proteins prevents hepatic lipid droplet accumulation and alters expression of genes involved in lipid metabolism in rats. Genes Nutr.

[8] Jang HH, Park MY, Kim HW, Lee YM, Hwang KA, Park JH (2012). Black rice (Oryza sativa L.) extract attenuates hepatic steatosis in C57BL/6 J mice fed a high-fat diet via fatty acid oxidation. Nutr Metab (Lond).

[9] Baur JA, Sinclair DA (2006). Therapeutic potential of resveratrol: the in vivo evidence. Nat Rev Drug Discov.

[10] Qureshi AA, Guan XQ, Reis JC, Papasian CJ, Jabre S, Morrison DC (2012). Inhibition of nitric oxide and inflammatory cytokines in LPS-stimulated murine macrophages by resveratrol, a potent proteasome inhibitor. Lipids Health Dis..

[11] Chen Q, Wang E, Ma L, Zhai P (2012). Dietary resveratrol increases the expression of hepatic 7alpha-hydroxylase and ameliorates hypercholesterolemia in high-fat fed C57BL/6 J mice. Lipids Health Dis..

[12] Signorelli P, Ghidoni R (2005). Resveratrol as an anticancer nutrient: molecular basis, open questions and promises. J Nutr Biochem.

[13] Di Pascoli M, Divi M, Rodriguez-Vilarrupla A, Rosado E, Gracia-Sancho J, Vilaseca M (2013). Resveratrol improves intrahepatic endothelial dysfunction and reduces hepatic fibrosis and portal pressure in cirrhotic rats. J Hepatol.

[14] Salado C, Olaso E, Gallot N, Valcarcel M, Egilegor E, Mendoza L (2011). Resveratrol prevents inflammation-dependent hepatic melanoma metastasis by inhibiting the secretion and effects of interleukin-18. J Transl Med..

[15] Zhang Y, Chen ML, Zhou Y, Yi L, Gao YX, Ran L (2015). Resveratrol improves hepatic steatosis by inducing autophagy through the cAMP signaling pathway. Mol Nutr Food Res.

[16] Zhu W, Chen S, Li Z, Zhao X, Li W, Sun Y (2014). Effects and mechanisms of resveratrol on the amelioration of oxidative stress and hepatic steatosis in KKAy mice. Nutr Metab (Lond)..

[17] Andrade JM, Paraiso AF, de Oliveira MV, Martins AM, Neto JF (2014). Resveratrol attenuates hepatic steatosis in high-fat fed mice by decreasing lipogenesis and inflammation. Nutrition.

[18] Chachay VS, Macdonald GA, Martin JH, Whitehead JP, O’Moore-Sullivan TM, Lee P, et al. Resveratrol does not benefit patients with nonalcoholic fatty liver disease. Clin Gastroenterol Hepatol. 2014;12(12):2092–2103 e2091-2096. 10.1016/j.cgh.2014.02.024 24582567

[19] Shen B, Yu J, Wang S, Chu ES, Wong VW, Zhou X (2008). Phyllanthus urinaria ameliorates the severity of nutritional steatohepatitis both in vitro and in vivo. Hepatology.

[20] Poulsen MM, Larsen JO, Hamilton-Dutoit S, Clasen BF, Jessen N, Paulsen SK (2012). Resveratrol up-regulates hepatic uncoupling protein 2 and prevents development of nonalcoholic fatty liver disease in rats fed a high-fat diet. Nutr Res.

[21] Jeon BT, Jeong EA, Shin HJ, Lee Y, Lee DH, Kim HJ (2012). Resveratrol attenuates obesity-associated peripheral and central inflammation and improves memory deficit in mice fed a high-fat diet. Diabetes.

[22] Xin P, Han H, Gao D, Cui W, Yang X, Ying C (2013). Alleviative effects of resveratrol on nonalcoholic fatty liver disease are associated with up regulation of hepatic low density lipoprotein receptor and scavenger receptor class B type I gene expressions in rats. Food Chem Toxicol..

[23] Scicchitano P, Cameli M, Maiello M, Modesti PA, Muiesan ML, Novo S (2014). Nutraceuticals and dyslipidaemia: Beyond the common therapeutics. Journal of Functional Foods..

[24] Faghihzadeh F, Adibi P, Rafiei R, Hekmatdoost A (2014). Resveratrol supplementation improves inflammatory biomarkers in patients with nonalcoholic fatty liver disease. Nutr Res.

[25] Pan QR, Ren YL, Liu WX, Hu YJ, Zheng JS, Xu Y (2015). Resveratrol prevents hepatic steatosis and endoplasmic reticulum stress and regulates the expression of genes involved in lipid metabolism, insulin resistance, and inflammation in rats. Nutr Res.

[26] Heeboll S, Thomsen KL, Clouston A, Sundelin EI, Radko Y, Christensen LP (2015). Effect of resveratrol on experimental non-alcoholic steatohepatitis. Pharmacol Res..

[27] Heeboll S, El-Houri RB, Hellberg YE, Haldrup D, Pedersen SB, Jessen N, et al. The effect of resveratrol on experimental non-alcoholic fatty liver disease depends on severity of pathology and timing of treatment. J Gastroenterol Hepatol. 2015; doi:10.1111/jgh.13151 10.1111/jgh.13151 26312773

[28] Singh R, Kaushik S, Wang Y, Xiang Y, Novak I, Komatsu M (2009). Autophagy regulates lipid metabolism. Nature.

[29] Hernandez-Gea V, Ghiassi-Nejad Z, Rozenfeld R, Gordon R, Fiel MI, Yue Z (2012). Autophagy releases lipid that promotes fibrogenesis by activated hepatic stellate cells in mice and in human tissues. Gastroenterology.

[30] Kim ES, Chang H, Choi H, Shin JH, Park SJ, Jo YK (2014). Autophagy induced by resveratrol suppresses alpha-MSH-induced melanogenesis. Exp Dermatol.

[31] Chang YP, Ka SM, Hsu WH, Chen A, Chao LK, Lin CC (2015). Resveratrol inhibits NLRP3 inflammasome activation by preserving mitochondrial integrity and augmenting autophagy. J Cell Physiol.

[32] Tanida I, Ueno T, Kominami E (2008). LC3 and Autophagy. Methods Mol Biol..

[33] Ichimura Y, Komatsu M (2010). Selective degradation of p62 by autophagy. Semin Immunopathol.

[34] Staels B, Rubenstrunk A, Noel B, Rigou G, Delataille P, Millatt LJ (2013). Hepatoprotective effects of the dual peroxisome proliferator-activated receptor alpha/delta agonist, GFT505, in rodent models of nonalcoholic fatty liver disease/nonalcoholic steatohepatitis. Hepatology.

[35] Henao-Mejia J, Elinav E, Jin C, Hao L, Mehal WZ, Strowig T (2012). Inflammasome-mediated dysbiosis regulates progression of NAFLD and obesity. Nature.

[36] Hassoun E, Mettling C. Dichloroacetate and Trichloroacetate Toxicity in AML12 Cells: Role of Oxidative Stress. J Biochem Mol Toxicol. 2015; doi:10.1002/jbt.21720 10.1002/jbt.21720 26121004

[37] Kleiner DE, Brunt EM, Van Natta M, Behling C, Contos MJ, Cummings OW (2005). Design and validation of a histological scoring system for nonalcoholic fatty liver disease. Hepatology.

